# Instruments to measure nurses' intention-to-stay in the profession: A systematic literature review

**DOI:** 10.1016/j.ijnsa.2026.100496

**Published:** 2026-01-26

**Authors:** Myrthe van der Zanden, Saba Hinrichs-Krapels, Christa Niehot, Anna Teeuw, Swasti Madan, Naomi van der Linden

**Affiliations:** a Institute for Health Systems Science; Faculty of Technology, Policy and Management; Delft University of Technology; Delft, The Netherlands; b Department of Internal Medicine; Section Nursing Science; Erasmus MC; University Medical Center Rotterdam; Rotterdam, The Netherlands; c Medical Library; Erasmus MC, University Medical Center Rotterdam; Rotterdam, The Netherlands; d Surgery Department; Dijklander Hospital; Hoorn, The Netherlands; e Independent researcher

**Keywords:** Nurses, Turnover, Retention, Intention to stay, Intention to leave, Workforce shortages

## Abstract

**Introduction:**

There is a large and increasing shortage of nursing staff. To alleviate this problem, healthcare systems should prioritize healthcare interventions that improve nurse retention over healthcare interventions that reduce it or leave it unchanged. One way to do so is to evaluate interventions on their anticipated impact on nurse intention-to-stay, which is an important precursor of retention. An overview of available instruments to quantify nurse intention-to-stay is lacking, resulting in researchers re-inventing the wheel. This review aims to fill this gap.

**Methods:**

A systematic literature search was performed in the databases Medline ALL via Ovid, Embase.com, Web of Science Core Collection, CINAHL Plus, PsycINFO, the Cochrane Central Register of Controlled Trials via Wiley, and Google Scholar (200 highest-ranked references only). The search string consisted of terms and associated synonyms for 1) nursing staff, 2) personnel intent to stay/leave, and 3) surveys. Articles were included when there was a quantitative method mentioned for measuring the intention of nurses to stay or quit nursing and/or their job/position/organization. Information was extracted on the year of publication, study design, study population, number of participants, instrument used for measuring intention-to-stay, and whether the instrument was focused on leaving the job, organization, or profession. In addition, we checked whether the instrument was used to evaluate the (expected or realized) impact of an intervention and if an association was determined between intention-to-stay (measured through the instrument) and retention. The protocol was not registered.

**Results:**

967 articles fulfilled our inclusion criteria, most of which were published in recent years. A total of 485 instruments were found. Nine regularly used instruments were identified, differing in their respective popularity over time, their size, the population for which they were developed and the strength of their link to actual retention. Notably, compared with the large body of literature on nurse intention-to-stay generally, the number of studies specifically measuring the impact of an intervention on nurse intention-to-stay is limited (n=20). Most of these intervention studies focused on changes in nurse training/mentorship or mental health support.

**Discussion & conclusion:**

Many different instruments exist to measure nurses' intention-to-stay. To add to our identified instruments, a comparative study is needed to identify which instrument offers the strongest predictive value for nurse retention. The absence of studies specifically evaluating the impact of interventions on nurses' intention-to-stay creates a critical gap in understanding how health interventions influence retention.

**Funding:**

Dutch Research Council, 406.XS.04.151.

**What is already known**

• Nurse turnover contributes to the large and increasing shortage of nursing staff.
• Healthcare interventions may increase or decrease nurse intention-to-stay.
• If interventions that increase intention-to-stay are prioritized, this may contribute to nurse retention.

Alt-text: Unlabelled box dummy alt text

**What this paper adds**

• Many different instruments are used to measure nurse intention-to-stay.
• Few studies measure the impact of an intervention on nurse intention-to-stay.
• It is unknown which instrument has the strongest predictive value for retention.

Alt-text: Unlabelled box dummy alt text

## 1 Introduction

The World Health Organization estimates a global shortage of 4.5 million nurses by the year 2030 (Boniol et al., 2022). Some factors driving this health workforce shortage are the aging population and a significant portion of healthcare professionals nearing retirement, with over one-third of doctors and a quarter of nurses in the European Union aged over 55 (OECD/European Commission, 2024). In the Netherlands, the issue is particularly severe, with projections indicating a shortage of up to 135,000 healthcare workers by 2031 (Kuffel, 2022). Additionally, nearly 40% of newly graduated nurses leave their positions or exit the sector entirely within two years, exacerbating the challenge of replacing those retiring or leaving for other reasons (Kox et al., 2020, Kuffel, 2022).

In the Netherlands, additional nurses are being trained, to reduce projected shortages. Capacity projections show that only part of these additional nurses, 35%, are needed to fulfill the growth in demand, while approximately 50% are needed to replace nurses who leave the profession for reasons other than retirement (Capaciteitsorgaan, 2022). Reasons for leaving the profession include lack of training, poor policy, planning, and human resources, lack of leadership management, increased workload, unattractive working conditions, and inadequate support (Tamata and Mohammadnezhad, 2023, de Vries et al., 2023).

Nurse turnover is costly for healthcare organizations and society (Bae, 2022, Duffield et al., 2014), and reduces the quality of care (Buchan et al., 2018, Bae, 2022) including patient safety (Zaheer et al., 2021). It is, therefore, not surprising that many researchers and healthcare organizations have tried to qualify and quantify nurse turnover. One way to do this is through surveys for nurses, asking them about their ‘intention’ to stay or leave. ‘Intention-to-stay’ and ‘intention-to-leave’ have been shown to precede actual retention/turnover (Al Yahyaei et al., 2022, Griffeth et al., 2000). Instruments that measure intention-to-stay or intention-to-leave can, therefore, qualify or quantify the extent to which a certain (sub)population of nurses is likely to leave their role or profession.

In order to improve nurse intention-to-stay and, therefore, retention, previous studies have explored job attributes that lead to nurse retention. Studies show that shared governance, autonomy in decision-making for nurses, and (technological) innovation can make the nursing job more attractive and improve retention (Duru and Hammoud, 2021, Hariyati and Nurdiana, 2019, Mohammadnejad et al., 2023). Nurse job attributes can be targeted by specific interventions aimed at improving retention, such as self-rostering to improve job satisfaction and autonomy (Leineweber et al., 2016, Webster and Archibald, 2022). In this paper, we call these ‘employee interventions’. However, other healthcare interventions -not specifically targeted at nurses- may also impact nurse intention-to-stay. For example, the organisation of care (e.g. concentration of care in a limited number of specialty centres) or the use of technology in care (e.g. telemonitoring, or care robots), may substantially impact the nursing job (Huter et al., 2020, Mohammadnejad et al., 2023) and therefore nurse intention-to-stay. In this paper, we call these ‘healthcare interventions’. For instance, the introduction of a new hospital-wide electronic health record (EHR) system to improve patient safety and care coordination is a healthcare intervention. A dedicated training program and workflow redesign to empower nurses in using that same EHR system would constitute an employee intervention.

Given the size of the current and future nursing shortages, there is a need for tools that enable healthcare systems to prioritize healthcare interventions that improve nurse retention over healthcare interventions that reduce it or leave it unchanged. However, in order to do so, we need to measure to what extent interventions impact retention or, its precursor, intention-to-stay. While the impact of an intervention on retention is sometimes captured in an observational study on the effectiveness of the intervention (e.g. in (Lee et al., 2000)), this is uncommon. The same is true for the impact of interventions on intention-to-stay (see section ‘interventions’ below). Ideally, one would be able to measure the *anticipated* impact of interventions on intention-to-stay in a certain nurse population/setting, before implementing the intervention. This would allow decision-makers to prioritize the right interventions, and move carefully in case of interventions that might reduce intention-to-stay.

Nurse intention-to-stay can be measured in various ways and, as far as the authors are aware, there is no overview of the instruments available to quantify it. In this systematic literature review, we provide an overview of the various instruments measuring intention-to-stay, and their use over time in nursing populations. We identify the advantages and disadvantages of the various instruments, including any evidence on the correlation between the instruments’ outcome and actual retention and/or turnover. In addition, we flag any cases in which an intention-to-stay instrument was used to evaluate the impact of an intervention, and whether a statistically significant change in intention-to-stay was observed.

Our aim is to provide an overview of available instruments suitable to quantify nurse intention-to-stay. This is a first step towards developing a standardized, validated approach to evaluate healthcare interventions on their anticipated impact on nurse intention-to-stay, to help reduce the nurse shortage.

## 2 Materials and methods

### 2.1 Search method

A search strategy was developed by an information specialist [CN] in collaboration with the study leads [MvdZ + NvdL]. The search was developed in Embase.com, optimized for sensitivity, and then translated to other databases following the method as described by Bramer et al.(Bramer et al., 2018). The search was carried out in the databases Medline ALL via Ovid (1946 to Daily Update), Embase.com (1971-present), Web of Science Core Collection (1975-present), CINAHL Plus (1982-present), PsycINFO (1806-present) and the Cochrane Central Register of Controlled Trials via Wiley (1992-present). Additionally, a search was performed in Google Scholar from which the 200 highest-ranked references were downloaded using the software Publish or Perish (Harzing, 2007). After the original search was performed in January 2024, the search was last updated on 12 November 2024.

The search strategies for Medline, Embase, CINAHL and PsycINFO used relevant thesaurus terms from Medical Subject Headings (MeSH), Emtree, CINAHL Subject Headings and PsycINFO respectively. In all databases, terms were searched in titles, abstracts, and author keywords. The search contained terms for 1) nursing staff, 2) personnel intent to stay/leave and 3) surveys, see the Supplement for the full search strategies. Terms were combined with Boolean operators (AND/OR) and proximity operators were used to combine terms into phrases. The full search strategies of all databases are available in the supplementary materials. The searches in Embase and Web of Science were limited to exclude conference papers. No study registries were searched, but Cochrane CENTRAL retrieves the contents of ClinicalTrials.gov and the World Health Organization's International Clinical Trials Registry Platform. No authors or subject experts were contacted and we did not browse unindexed journals in the field.

The references were imported into EndNote and duplicates were removed by the information specialist [CN] using the method as described by Bramer et al (Bramer et al., 2016).

Two reviewers [MvdZ + NvdL] manually screened the title/abstract using Covidence. After screening the title/abstract, full text screening was done in Covidence by the same researchers [MvdZ + NvdL]. Any discrepancies in the verdict were resolved by discussion between the two reviewers [MvdZ + NvdL].

A PROSPERO review protocol was not created prior to this systematic literature review due to the nature of the review not meeting PROSPERO’s criteria. Specifically, the review did not involve pre-specified interventions or measurable outcomes, which are required for PROSPERO registration. As a result, it was not possible to comply with the necessary requirements for protocol registration.

### 2.2 Inclusion and exclusion criteria

Articles were included when there was a quantitative instrument mentioned for measuring the intention of nurses to stay or quit nursing and/or their job/position. Both nurse ‘intention-to-stay’ and ‘intention-to-leave’ instruments were included. When mixed-methods were used, and intention-to-stay or leave was measured quantitatively, the study was included as well. Protocol papers were also included to capture planned uses of instruments, signaling the direction of this research area. Further inclusion criteria were: nurses in any grade/level, nurse aids, and nurse assistants, working in all settings in healthcare. Papers were excluded if a full text version was not available, or if the paper was written in another language than English or Dutch. Another exclusion criterion was when the intention-to-stay was not measured in nurses, but in a different or in a mixed population. For example, we did not include papers on physicians, nurse managers or student nurses. Therefore, instruments used in such papers were only captured if they were also used in a population of (only) nurses. There were no limits to year of publication.

### 2.3 Quality appraisal

The methodological quality of identified studies was assessed only for the pivotal publications that originally developed or first introduced each instrument (see Table 1), using the COSMIN Risk of Bias checklist (Mokkink et al., 2018). No further quality appraisal was conducted for the remaining studies, as the primary objective of this review was to identify which measurement instruments have been used to assess nurses’ intention-to-stay, rather than to evaluate effect sizes. Moreover, the quality of a study applying an instrument cannot be used as a proxy for the measurement properties of the instrument itself.

**Table 1:** Regularly used instruments in nurse intention-to-stay research.

Instrument	Number of items	Measurement scale	Originally developed in the population	Cronbach’s alpha in original study	Cronbach’s alpha range	Relation to actual turnover
Anticipated Turnover (Hinshaw and Atwood, 1984)	12	7 (original), 6 and 5-point Likert scales have been used	Hospital nurses, USA	0.84	0.70 ((Cheng and Liou, 2011), 12 items, 7-point scale) - 0.94 ((Hart, 2005), 12 items, scale NR)	Accurate predictor for actual turnover in Wagner 2010 (R^2^ = 0.255, p<0.0005) (Wagner, 2010).
Michigan Organization Assessment Questionnaire (Cammann et al., 1983)	3	7, 5 (original), and 4-point Likert scales have been used, with the 7-point scale being most common	Employees, USA	0.83	0.69 ((Krausz et al., 2000), 3 items, 5-point scale) - 0.95 ((Sungur et al., 2019), 3 items, 5-point scale)	Spearman correlation of 0.81 (n.s.) between turnover intention at a certain measurement point (“time 2”) and actual turnover (Gaudine and Thorne, 2012).
Voluntary turnover intention scale (Mobley et al., 1978)	3 – 13 (depending on the version, originally 3)	5-point Likert scale (incidentally adapted to 7-point)	Hospital employees, USA	0.90	0.66 ((Kim et al., 2024), 6 items, 5-point scale) - 0.947 ((Lo et al., 2023), 3 items, scale NR)	See main text for adjusted odds ratios for the various intent-to-leave subscales.
Turnover intention scale (Michaels and Spector, 1982)	3 – 6 (depending on the version, originally 3)	6, 5 and 4-point Likert scales have been used (original paper does not report scale)	Mental health facility employees, USA	0.87 ((Lee et al., 2011))	0.75 ((Chen et al., 2023), 6 items, 4-point scale) - 0.95 ((Zhang et al., 2024), 6 items, 4-point scale)	Correlation with actual turnover: r = 0.41.
Turnover intention scale (Kelloway et al., 1999)	3 – 4 (originally 4)	5-point Likert scale	Employees from hospitals and grocery stores, Canada	0.92	0.80 ((Karimi Rozveh et al., 2023), 4 items, 5-point scale) -0.94 ((Nikkhah-Farkhani and Piotrowski, 2020), 4 items, 5-point scale)	NR
McCain’s Intent to Stay Scale (McCloskey, 1990)	4 - 5 (part of a larger, 38-item scale, originally 5 ITS items)	5-point	Newly employed hospital nurses	0.88	0.73 in the public hospitals sample ((AbuAlRub et al., 2009), 5 items, 5-point scale) – 0.93 ((Mrayyan, 2007), 5 items, 5-point scale)	NR
Turnover inventory scale (Bothma and Roodt, 2013)	6	5-point Likert scale. 4-point has also been used (Li et al., 2020)	Employees in an ICT company	0.80	0.73 ((Smama’h et al., 2023), 6 items, 5-point scale) – 0.88 (for an adjusted version by (Liu et al., 2019) 6 items, 5-point scale, reported in (Holter, 2022))	NR
Turnover intention measured with Park's tool (Park, 2002)	4 (a 7-item version has also been used, (Minkyung et al., 2022))	5-point Likert	Employees from nursing organizations (Mohammadnejad et al., 2023)	0.88	0.77 ((Kim et al., 2019), 4 items, 5-point scale) – 0.93 ((Yang and Kim, 2016), 4 items, 5-point scale)	NR
Nurse retention index (Cowin, 2002)	6 – 8 items (originally 8)	8-point Likert 6-point Likert has also been used (Choi and Lee, 2023)	Nurses in Australia	0.97	0.91 ((Kim and Lee, 2023), 6 items, 8-point scale), -0.96 ((Choi and Lee, 2023), 8 items, 6-point scale)	NR

Abbreviations: ICT = information and communication technology, NR = not reported, USA = United States of America.

### 2.4 Data extraction/synthesis and analysis

A data extraction sheet was developed to extract relevant information, including year of publication, study design, study population, number of participants, instrument used for measuring intention-to-stay, and whether the instrument was focused on leaving the job, organization or profession. In addition, we checked whether the instrument was used to evaluate the (expected or realized) impact of an intervention and if an association was reported between the outcome of the instrument and actual retention. The frequency of use of each instrument was also recorded, along with a distinction between studies that involved interventions and those that did not.

Data was extracted by the researchers and a research assistant [MvdZ + NvdL + SM]. The data extraction process was performed in a systematic manner to ensure consistency and reliability. Each study was reviewed independently by a researcher and a research assistant to extract the relevant data points. This process involved cross-checking for any inconsistencies or missing data, and where necessary, resolving discrepancies through discussion among the research team. The extracted data was then systematically organized and summarized to provide insights into the patterns of instrument use across different studies.

The information was visualized through tables and Fig.s, offering an accessible overview of key trends and findings. These visual representations helped to further clarify the frequency of use per instrument, their focus areas (e.g., job, organization, or profession), and the relationship between intention-to-stay measures and actual retention outcomes.

## 3 Results

### 3.1 Search results

**Fig. 1:**
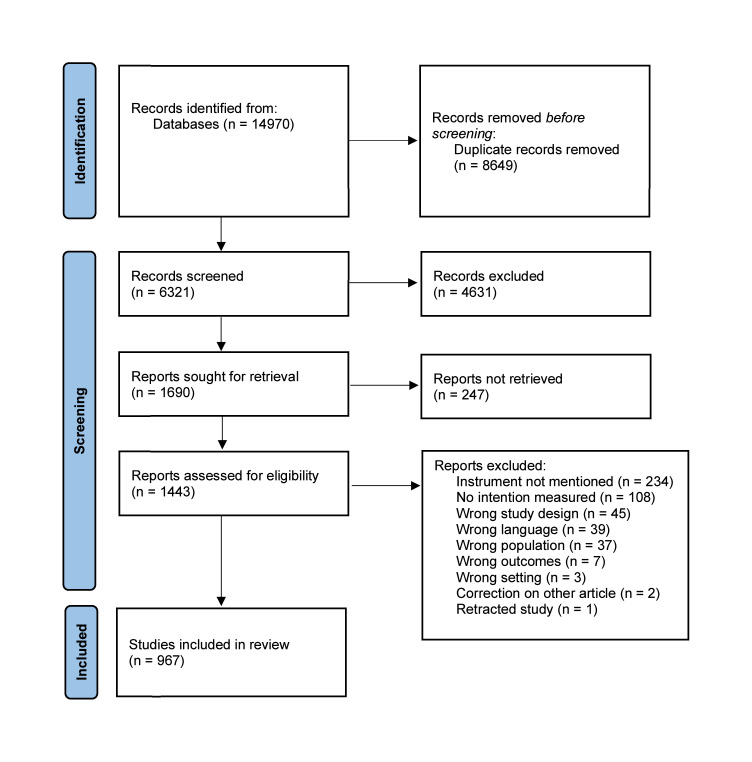
PRISMA flow chart.

After deduplication 6,382 records were identified in the database search. After duplicate removal, 6,321 studies were screened by title and abstract, from which 1,690 were reviewed as full-text articles. Finally, 967 articles fulfilled the inclusion criteria, and data were extracted. The study selection process is shown in the flow chart (Fig. 1).

### 3.2 Study characteristics

**Fig. 2:**
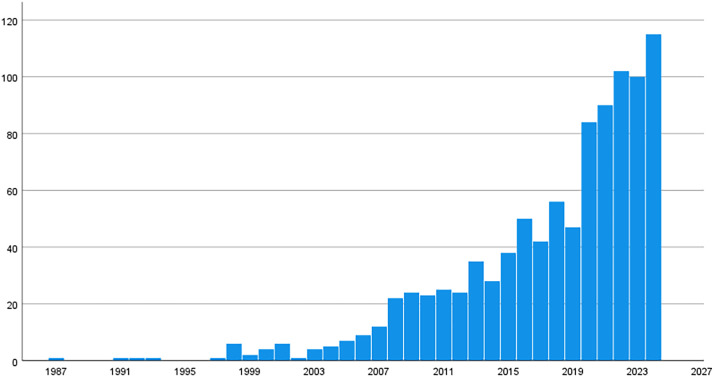
Number of publications about intention to stay per year.

Included studies were published between 1987 and 2024. The number of studies fulfilling our inclusion criteria has increased significantly in recent years, with a notable rise beginning around 2008 (see Fig. 2). The peak is observed in the years 2020 and beyond, with over 100 studies published in 2023, and 115 studies published in 2024.

Due to study protocols being included, the number of nurses included per study ranges from 0 (study protocol) to 207.6363 nurses [IQR 212 – 789]. The studies were conducted across various countries, with notable contributions from China, Ghana, Saudi Arabia, the Philippines, and the United States, highlighting a global interest in understanding nurses' turnover intentions. The majority of studies were performed in the hospital setting, ranging from studies in a single hospital department (e.g., pediatric or intensive care units) to large, multi-department, and multi-center hospital studies. Some studies also examined other settings such as nursing homes, public healthcare facilities, or mental health services.

### 3.3 Overview of included instruments

**Fig. 3:**
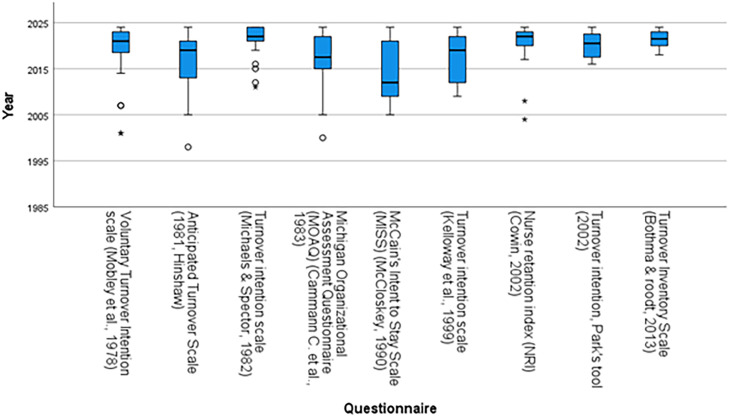
Boxplot of top 9 questionnaires used over the years, sorted by year.

In total, 485 different quantitative instruments were used to measure intention-to-stay or similar constructs such as intention-to-leave. Most articles (N= 375) used a self-developed instrument. While self-developed instruments allow researchers to phrase their question(s) specifically with their aim and target population in mind, it limits opportunities for comparisons across studies, and prevents researchers from expanding the evidence base on previously developed instruments. A total of 593 studies used a pre-developed instrument. The nine most commonly used instruments were the Anticipated Turnover Scale (N=46)(Hinshaw and Atwood, 1984), Michigan Organizational Assessment Questionnaire (N=42) (Cammann et al., 1983), the Voluntary Turnover Intention scale (N=41) (Mobley et al., 1978), the Turnover intention scale (N=37) (Michaels and Spector, 1982), the Turnover intention scale (N=25) (Kelloway et al., 1999), the Turnover inventory scale (N=18) (Bothma & Roodt, 2013), McCain’s Intent to Stay Scale (MISS) (N=17) (McCloskey, 1990), the Nurse retention index (NRI) (N=16)(Cowin, 2002), and Park’s Tool (N=17)(Park, 2002), see Fig. 3.

Table 1 below reports on the main characteristics of identified, regularly used intention-to-stay instruments in nursing research. Quality assessment showed that most pivotal publications on these intention-to-stay instruments emphasized internal consistency and validity of the instrument over stability. Using COSMIN, all nine instruments scored ‘inadequate’ on test-retest reliability and measurement error, as no repeated-measures data were reported. While most scales showed strong internal consistency in at least some published papers (see Table 1), stability evidence was not provided in the included studies.

The following sections will provide more information about each of the five most regularly used instruments, to help researchers select the instrument most relevant to their research. All but one of these instruments focus on leaving the current position. The turnover intention scale by Kelloway et al., 1999 considers leaving the organization as a whole, instead of the current position. No instrument considered leaving the nursing profession as a whole.

### 3.4 Anticipated Turnover Scale (Hinshaw and Atwood, 1982)

The Anticipated Turnover Scale from Hinshaw et al. (Hinshaw and Atwood, 1982), developed in 1978, is the most commonly used intention-to-stay-instrument in nursing research (N=46). The instrument was originally tested amongst nurses in 15 urban and rural hospitals throughout Arizona and has since been used in many different countries and settings. The 12 questions, both positively and negatively phrased to avoid bias, address two dimensions of nurses’ perceptions of voluntarily leaving: initial expectations of staying in the position, and certainty of anticipated leaving (Wagner, 2010).

Questions are answered on a 7-point Likert scale, resulting in a score between 12 and 84 (ref Hauck 2011). Higher scores reflect greater intent to leave the present position or job: 12-24 is considered low, 25-42 moderate and 43-84 high turnover intention (Al Zamel 2021). Versions with a 6-point or 5-point Likert scale have also been used (Cheng et al., 2014, Shoorideh et al., 2015).

The Anticipated Turnover Scale was a reasonable predictor for actual turnover (R^2^ = 0.255, p<0.0005) (Wagner, 2010). Cronbach’s alpha was 0.84 in the original study, and ranged between 0.70 (Cheng 2011) and 0.94 (Hart 2005) in later studies. Barlow and Zangoro (Barlow and Zangaro, 2010) conducted a meta-analysis to determine the consistency of reliability estimates and evidence of construct validity of the Anticipated Turnover Scale scores across nursing studies in the United States of America. The overall mean weighted effect size of reliability from 12 studies (n = 2,442) was 0.89, indicating excellent reliability and construct validity (Almalki et al., 2012).

While developed in English, many translations of the Anticipated Turnover Scale are available, such as a Persian version (Rivaz et al., 2021), a Chinese version (Liu and Aungsuroch, 2018), a Korean version (Nam et al., 2021), a Bahasa Malaysian version (Al Zamel et al., 2021) and a Portuguese one (de Sul and Lucas, 2020).

### 3.5 Michigan Organization Assessment Questionnaire (CAMMANN et al., 1983)

The Michigan Organizational Assessment Questionnaire was originally developed to study organizational attitudes and behaviors across a broad range of employees, not specific to nurses or the healthcare sector. Subsequently, it has been often used for nurses, including hospital nurses (İspir Demir et al., 2023, Gherman et al., 2022, Chien and Yick, 2016), mental health nurses (Duncan, 2024) and nursing home nurses (Ausserhofer et al., 2023), amongst others.

The questionnaire includes three intention-to-stay items, addressing how likely respondents are to leave their current position within the year, whether they think about leaving, and how likely it is that they could find a job with another employer with the same pay and benefintention-to-stay (Armmer and Ball, 2015). A higher score indicates that nurses are more likely to leave their current organization and are more likely to seek another job in the coming year (İspir Demir et al., 2023). In various studies, the first item was modified from the form of a question, asking about one’s likelihood of looking for a new job, to a statement that follows the item structure of the other two items (Glazer and Kruse, 2008). 4-point Likert (Duncan, 2024), 5-point Likert (Ausserhofer et al., 2023) and 7-point Likert scales have been used (İspir Demir et al., 2023, Gherman et al., 2022, Chien and Yick, 2016).

The originally reported Cronbach alpha was 0.83 (Simons, 2008). In later studies in nurses, Cronbach’s alpha ranged between 0.69 (Krausz et al., 2000) and 0.93 (Filipova, 2011). Cramer et al. found that instrument coefficient alpha’s were greatly improved when the third item of the scale was removed; thus, the score was computed from an average of 2 of the 3 items. They explain: “It might be conjectured that the third item of the scale that was removed (“How likely is it that you could find a job with another employer with about the same pay and benefits you have now?”) may reflect more on the profession of nursing itself. In other words, it may not have the same assumed relationship to the first two variables that it does for the other professions for which this scale was developed.”(Cramer et al., 2014).

Gaudine et al. found a Spearman correlation of 0.81 (n.s.) between turnover intention at a certain measurement point (“time 2”) and actual turnover (Gaudine and Thorne, 2012).

The instrument was developed in English. A Turkish version of this instrument is also available (İspir Demir et al., 2023).

### 3.6 Voluntary turnover intention scale (Mobley et al., 1978)

This turnover intention scale was developed by Mobley et al. (Mobley et al., 1978), and later modified and supplemented by various authors, such as Moon and Han (Moon and Han, 2011). The original work by Mobley et all focused on hospital employees in the United States of America. The scale primarily includes items for measuring employees’ dissatisfaction with their organization or hospital, employees’ consideration to leave their current jobs, and, in some versions, the possibility of finding a job (Yeh et al., 2020, Han et al., 2015). Other authors describe the scale as capturing three phases of the process: thinking about leaving, thinking about job searching, and searching for a job (Castle et al., 2007).

Substantial variability exists between different versions of the questionnaire. The majority of studies use versions with 3 to 6 items, but versions with 7 items (Crowe et al., 2022) or even 13 items (Baek and Lee, 2022) have also been used. When using the 3-item version and 5-point Likert scale, scores range between 3 and 15, with higher scores indicating a higher intention to change jobs (Naserian et al., 2024). A 7-point Likert scale has been used by Li et al (Li et al., 2021).

The original scale showed adequate construct validity and a Cronbach’s alpha of 0.90 (Mobley et al., 1978). In later studies, Cronbach’s alpha ranged between 0.66 ((Kim et al., 2024), six items) and 0.947 ((Lo et al., 2023), three items), meaning that the internal consistency may differ between settings. However, Cronbach’s alpha is also dependent on the number of items, as it increases with a higher number of items, even if the average inter-item correlation remains the same.

Castle et al. used the instrument to measure nurse aides' intention-to-leave in nursing homes (Castle et al., 2007). This study reported adjusted odds ratios for the three intent-to-leave subscales, with actual turnover after one year as dependent variable: 0.80 (95%CI 0.55-1.02) for thinking about leaving, 0.95 (95%CI 0.72-1.03) for thinking about job search, and 1.05 (95%CI 1.00-1.20) for searching for a job (Castle et al., 2007).

While developed in English, various translated versions have been developed, including a Chinese version (Hong et al., 2023) and a Turkish one (Kucukkelepce, 2022). The instrument has also been used to measure the emigration intentions of specialized nurses in Ghana (Poku et al., 2023). Hackett et al. used the instrument both for measuring organizational withdrawal intentions and for measuring occupational withdrawal intentions by replacing ‘job’ with ‘career’ across all items (Hackett et al., 2001).

### 3.7 Turnover intention scale by (Michaels & Spector 1982)

This instrument was developed by Michaels & Spector (1982) as part of a study testing the ‘Mobley, Griffeth, Hand, and Meglino turnover model’ in the United States (Michaels and Spector, 1982). It was originally administered to employees of a mental health facility, but has now demonstrated wide applicability across various populations and settings (Liu et al.), including Asian nurses from various specialties (e.g. psychiatry, emergency care) in South-Korea and China.

A range of names is used to refer to this instrument, including the ‘intention of quitting scale’ (Lee et al., 2012), the ‘turnover intention scale’ (Ma et al., 2022, ALLAN and RAYAN, 2023, Liu et al.), the ‘turnover intention questionnaire (TIQ)’ (Wang et al., 2022) and the ‘Nurse Turnover Intention Scale (NTIS)’ (Hu et al., 2022).

Different versions of this instrument exist, in different languages and with either a 4-point Likert scale (Li et al., 2019, Li et al., 2024), 6-point Likert scale (Ali and Rayan, 2023), or using a visual analogue scale (Lee et al., 2012). Three dimensions are measured, through 3-6 questions, depending on the version. The dimensions address (I) the possibility of resignation from the present job, (II) the motivation to seek other jobs, and (III) the possibility of obtaining an external job (Li et al., 2019). In many publications using this instrument, the total score was calculated, but also separate scores for each of the three dimensions (Li et al., 2019).

Range in total score depends on the version used, e.g. 6-24 for the 4-point version with 6 questions, or 0-30 for the VAS-version. Some studies have worked with classification of responders in three or more groups, e.g. to minimize the effects of neutral responses (Lee et al., 2011). Turnover intention measured through this instrument was correlated with actual turnover, r = .41 (Michaels and Spector, 1982). Cronbach’s alpha ranged between 0.70 (Chen et al., 2023, Su, 2021) and 0.95 (Zhang et al., 2024).

### 3.8 Turnover intention scale (TIS) by (Kelloway et al. 1999)

The TIS by Kelloway et al. measures thoughts about leaving the current organization and seeking job opportunities (Foster et al., 2024). It was originally developed in employees from healthcare and a retail grocery organization. As a result, nursing and in-store personnel (e.g. cashiers, stock takers) were the most frequently occurring occupations in the sample (Kelloway et al., 1999). The TIS uses four items, measured on a 5-point Likert scale, resulting in a score between 4 and 20. Higher scores indicate a higher turnover intention. Some studies used three instead of four items of this scale (Favaro et al., 2021).

The original studies found this instrument to be a reliable measure with high internal consistency (Cronbach's alpha = 0.92–0.93; (Kelloway et al., 1999) in (Foster et al., 2024). Cronbach’s alpha’s of subsequent studies lay between 0.80 (Karimi Rozveh et al., 2023) and 0.94 (Nikkhah-Farkhani and Piotrowski, 2020, Strunk and Strunk, 2012). Poku et al. used three of the four items, and found a Cronbach’s alpha of 0.92 (Poku et al., 2023).

The instrument was developed in English. It was translated for use in Iran (Shahpouri et al., 2016).

### 3.9 Interventions

As mentioned in the introduction, it may be possible to use intention-to-stay instruments to quantify the anticipated or realized impact of interventions on nurse intention-to-stay. Table 2 lists all 20 papers that reported on the impact of an intervention on intention-to-stay. Interventions included changes in employment conditions (n=2), organizational and process changes (n=3), changes in training and mentorship (n=8), and interventions related to mental health support, e.g. coping, mindfulness, or psychotherapy (n=6). We would classify all of these as ‘employee interventions’, as discussed in the introduction of this manuscript. Only 1 paper reported on the impact of an intervention on direct nursing tasks or patient care, namely the implementation of bedside handover (Malfait et al., 2019). This is what we would consider a ‘healthcare intervention’.

**Table 2:** Papers reporting on the impact of an intervention on intention-to-leave.

	Intervention	Instrument	Study design	N	Change
**Changes in employment conditions**					
De Groot et al. 1998 (De Groot et al., 1998)De Groot et al. 1998(De Groot et al., 1998)	Differentiated pay structure	Anticipated Turnover Scale (Hinshaw and Atwood, 1984)	Quasi-experimental	232	N.s.
Ahn & Choi 2023 (Ahn and Choi, 2023)	Career ladder system	Turnover Scale by Mobley et al. (Mobley et al., 1978)	Cross-sectional	274	N.s. for the difference in mean turnover intention between implementation and non-implementation groups
**Organizational and process changes**					
Lee et al. 2000 (Lee et al., 2000)	Quality circle program	1 self-developed question	Quasi-experimental	53	Lower ITL
Burke et al. 2000 (Burke and Greenglass, 2000)	Hospital restructuring and downsizing	2 self-developed questions	Cross-sectional	1362	NR
Breckenridge-Sproat et al. 2017 (Breckenridge-Sproat et al., 2017)	Patient Caring Touch System (various components)	1 self-developed question	Pre-post	483 pre + 660 post	Higher ITL (but there was a concurrent decline in staffing levels)
**Changes in training & mentorship**					
Newhouse et al. 2007 (Newhouse et al., 2007)	Internship program (Social and Professional Realitt Integration for Nurse Graduates)	Anticipated Turnover Scale (Hinshaw and Atwood, 1984) + actual retention	Quasi-experimental	522	Lower ITL
Bozionelos 2009 (Bozionelos, 2009)	Cross-cultural training	3 self-developed questions	Cross-sectional	206	N.s.
Wallen et al. 2010 (Wallen et al., 2010)	Structured, multifaceted mentorship programme on evidence-based practice	Intent to Leave Scale (Price and Mueller, 1983) and Nurses' Retention Index (Cowin, 2002)	Quasi-experimental	99	N.s.
Cramer et al. 2014 (Cramer et al., 2014)	Continuing education course to prepare nurses for national board certification and improve technological competence	Intent to Turnover Scale (Cammann et al., 1983)	Quasi-experimental	84	NR
Medas et al. 2015 (Medas et al., 2015)	Nurse residency program for newly licensed registered nurses	3 self-developed questions	Prospective cohort study	79	NR
Jones 2017(Jones, 2017)	Formal, 12-month nurse mentor program pilot (1-on-1 mentoring by experienced nurse)	Intention to Stay/Leave Job Diagnostic Survey (Hackman, 1974)	Pre-post	4	N.s. (after 3 months)
Gulsen et al. 2022 (Gülşen and Kutlu, 2024)	Four-week training program based on 'caring culture'	1 question based on Biegger et al., 2016 (Biegger et al., 2016)	Pre-post	86	Negative correlation between caring culture and intention to leave
Song et al. 2024 (Song et al., 2024)	Nurse residency program including hospital orientation, common curriculum education, preceptorship, educational mentoring and an educational nursing supporting program	Turnover intention measurement tool by Lawler (1983) and adapted by Park (Park, 2002)	Quasi-experimental	167	Lower ITL and lower turnover
**Mental health support**					
Guo 2015 (2018)	Three good things' positive psychotherapy	Turnover intention scale (Michaels and Spector, 1982)	RCT (study protocol)	73	NR (study protocol)
Kang et al. 2017 (Kang et al., 2017)	Cognitive rehearsal program	Yun's turnover intention scale(Yun and Kang, 2018)	RCT	40	Lower with intervention
Kang et al. 2019 (Kang and Jeong, 2019)	Smartphone application to cognitively train nurses to handle bullying situations in the workplace	Intent to leave instrument, becker (1992)	Cluster quasi-randomised trial	72	Lower with intervention
Maatouk 2018 (Maatouk et al., 2018)	Intervention programme with exploration of professional biography, awareness and relaxation training, SOK modules (aging strategies).	1 question based on thinking of giving up the nursing profession (Maatouk et al., 2018)	Pre-post	115	NR
Rusthon et al. 2021 (Rushton et al., 2021)	Mindful Ethical Practice and Resilience Academy (MEPRA)	A 1-item question measured turnover intentions: “In the past week, I have seriously thought about looking for a new job.” [1 (strongly disagree) to 5 (strongly agree)] Ref: Bothma CFC, Roodt G. The validation of the turnover intention scale. S A J Hum Resource Manage. 2013;11(1):1-12.	Pre-post	415	N.s. (trend towards statistically significant decrease)
Foster et al. 2024 (Foster et al., 2024)	Resilience-building programme delivered by trained facilitators across two workshops	Turnover intention scale by Kelloway et al. 1999 (Kelloway et al., 1999)	Clustered RCT	144	N.s.
**Changes in nursing tasks/patient care**					
Malfrait et al. 2019 (Malfait et al., 2019)	Bedside handover	Turnover intention scale (Cammann et al., 1983)	Longitudinal	165	N.s.

Abbreviations: ITL = intention-to-leave, n.s. = not significant, NR = not reported, RCT = randomised controlled trial.

Only five studies found a reduction in intention-to-stay associated with their interventions: a quality circle program in Lee et al. 2000 (Lee et al., 2000), an internship program in Newhouse et al. 2007 (Newhouse et al., 2007), a cognitive rehearsal program in Kang et al. 2017 (Kang et al., 2017) and Kang et al. 2019 (Kang and Jeong, 2019) and a nurse residency program in Song et al. 2024 (Song et al., 2024). It is impossible to compare outcomes between these interventions, because different intention-to-stay instruments and study designs were used in each of these studies.

While only one included paper addressed a ‘healthcare intervention’, several papers (which were not included in Table 2) alluded to healthcare interventions that may impact intention-to-stay. For example, one paper reported on the association between the usability of electronic health records and intention-to-leave (Kutney-Lee et al., 2021). This study was not included in Table 2 since it did not report on the effect of an intervention, however, it did find statistically significant higher odds of intention-to-leave with poor usability of electronic health records. This suggests that increasing the usability of electronic health records may reduce the intention-to-leave.

Similarly, another paper reported on the association between administrative task burden and intention to leave the profession, for nurses in 118 Swiss nursing homes. Nurses reporting higher administrative task burden were more likely to intend to leave the profession (OR=1.24; 95%CI: 1.02-1.50), suggesting a lower administrative task burden may reduce intention-to-leave (Ausserhofer et al., 2023).

## 4 Discussion

### 4.1 Findings

A large body of literature is available on nurse intention-to-stay. In total, 485 instruments to measure nurse intention-to-stay have been identified in this literature review. These include single self-developed questions but also extensive questionnaires. Holter (Holter, 2022) previously noted: “Several methods of measuring turnover intention exist, however, there are few validated scales, many researchers have resorted to a single question about turnover, which has been criticized as a true method for measuring turnover intention”. The oldest paper mentioning the use of an intention-to-stay instrument in a nursing population, picked up by our search strategy, is from 1987. Since 2002, the number of nurse intention-to-stay-studies published per year has steadily increased to 115 in 2024.

Compared with the large body of literature on nurse intention-to-stay, the number of studies measuring the impact of an intervention on nurse intention-to-stay is limited. Only 20 intervention studies were identified, many of which focused on interventions changing the way nurses function in or cope with (challenging aspects of) their work, instead of changing (such challenging aspects of) their work or work conditions. There was no consistency in the intention-to-stay measures used in these various studies on interventions. Also, information is lacking on the impact of healthcare interventions impacting nurse intention-to-stay, as opposed to employee interventions. For example, the authors wonder about the impact of technology (e.g. care robots, telemonitoring) on nurse intention-to-stay, and the impact of system-level interventions (e.g. centralisation of care) on outcomes such as nurse intention-to-stay. Research on this is lacking.

### 4.2 Choice between instruments

We selected the nine most commonly used instruments for further evaluation. These tools differ in popularity over time, length, response format, the populations in which they were developed and the strength of their link to actual retention. The choice between them should therefore depend on the nursing population under study, the need for brevity versus detail, and other practical requirements. Comparative research remains necessary to determine whether any instrument consistently outperforms the others, particularly in predicting actual turnover.

The Anticipated Turnover Scale by Hinshaw and Atwood is the most widely used in nursing research. It provides a comprehensive measurement, but its 12-item length can be a drawback in large-scale surveys. Importantly, it is also one of the few instruments with demonstrated predictive validity for actual turnover, making it a strong option when accuracy is prioritized. However, its phrasing is largely generic, raising questions about whether it captures nursing-specific concerns more effectively than other scales. This issue applies broadly, as none of the available instruments are exclusively tailored to nursing populations.

Shorter scales, such as the Michigan Organization Assessment Questionnaire and the Turnover Intention Scales by Michaels & Spector and Kelloway et al., offer efficiency. The Nurse Retention Index, developed specifically for nurses, combines brevity with very high internal consistency, yet evidence of its link to actual turnover is lacking. Other instruments, including Mobley’s Voluntary Turnover Intention Scale and McCain’s Intent to Stay Scale, provide flexibility or nursing-oriented phrasing but are similarly underexplored in terms of predictive validity.

Beyond length and reliability, the original population in which each tool was developed should be considered. Several scales were designed for general employee groups rather than nurses, which may limit their sensitivity to the unique pressures of nursing work. Even healthcare-focused instruments often rely on generic wording, reducing their ability to capture sector-specific concerns. Moreover, while most scales demonstrate acceptable internal consistency, data on stability and association with actual turnover is lacking, underscoring the need for validation studies.

Finally, it is important to recognize that these instruments primarily assess cognitive decision-making processes. In doing so, they may overlook impulsive or emotionally driven turnover behaviors, which are increasingly relevant in high-stress environments. Reliance on self-reported data also introduces potential bias, as responses can be shaped by temporary emotions or social desirability. External factors such as job market conditions, financial constraints, and cultural differences further complicate the link between intention-to-stay and actual retention. These limitations highlight the importance of selecting instruments carefully and interpreting results within the broader organizational and social context.

### 4.3 Strengths and limitations

To our knowledge, this is the first systematic literature review on nurse intention-to-stay instruments. We believe it provides a valuable overview of available instruments so that researchers in this area can make a better-informed decision on the most suitable instrument to include in their own studies. Given the societal importance of reducing nurse turnover, we expect the number of studies in this area to continue to grow. If a larger proportion of these studies use proven, validated intention-to-stay questionnaires appropriate to the population of interest, they will make a stronger contribution to the evidence base that may help reduce nurse turnover.

This review only identified studies using an intention-to-stay instrument in a nursing population, irrespective of which population it was developed for. Other valuable intention-to-stay instruments exist, and may also be appropriate for use in nurses. We decided not to include (studies on) these instruments since the amount of data on instruments specifically used in nurses was already extensive and provided insight into the attributes of these instruments for the population of interest. Intention-to-stay instruments developed in other (health or non-health) professions, may not have the same characteristics (e.g. reliability, validity) in a nursing population.

While we mention the population in which each of the intention-to-stay instruments was developed, including the setting (hospital care, mental healthcare, community care, etc.), we did not discuss these settings separately. An instrument that is developed in hospital nurses, may not have the same characteristics in other types of nurses. For example, the wish to leave a position may have a stronger correlation with actual turnover in settings in which it is easy to move between positions, e.g., due to large shortages or good opportunities for intra-organizational mobility.

Additionally, the lack of proactive protocol registration is acknowledged as a limitation. However, the study methods were pre-specified and all reported outcomes align with the initial objectives. Please also note that we only included publications written in English or Dutch. We will have missed publications in other languages, and therefore potentially intention-to-stay instruments developed in countries where it is less common to publish in English. This language restriction may have introduced cultural bias, limiting the generalizability of our conclusions.

Another limitation is that we relied on published journal articles and did not include grey literature (e.g., dissertations, reports, conference proceedings). This may have led to publication bias, as studies with less favourable or inconclusive findings are less likely to be published in peer-reviewed journals. Study protocols were included to capture planned uses of instruments, strengthening the analysis by reflecting the future direction of the field.

Finally, our search strategy, while comprehensive, may still have missed relevant studies due to differences in indexing, terminology, or incomplete reporting of instrument use. This could have led to selection bias and an incomplete overview of available instruments.

### 4.4 Future Research Directions

Building on these limitations, future research should:

• prioritize head-to-head, comparative validation studies of the most promising instruments to directly assess the predictive validity of different intention-to-stay instruments across diverse nursing populations and care settings. Such work would help researchers choose between instruments, and would also help determine whether instruments developed in one context (e.g., hospital nurses) are equally robust in others (e.g., community or mental health nurses).
• develop and validate a short, generic module for intention-to-stay that can be incorporated into broader evaluations of healthcare system changes.
• prioritize the use of such a module in intervention studies, particularly for evaluating ‘healthcare interventions’ (e.g., technological implementations, process re-design) for which impact on the nursing workforce is currently unknown.
• further explore and quantify the impact of healthcare interventions (rather than employee interventions only) on nurse intention-to-stay.

In addition, cross-cultural adaptation studies could ensure that intention-to-stay instruments capture culturally specific factors influencing nurse retention.

## 5 Conclusion

Many different instruments are available to measure nurse intention-to-stay. Some of these, such as the Anticipated Turnover Scale (Hinshaw and Atwood, 1984) and the Turnover intention scale (Michaels and Spector, 1982), were shown to correlate reasonably well with nurse turnover. If we want to reduce nurse turnover, we need to understand which interventions would have the greatest impact on retention. The impact of such interventions on intention-to-stay should be quantified using a validated and suitable intention-to-stay instrument so that the results of such an evaluation can inform decision-making, e.g., by hospital management, health insurers, intervention developers, etc. This would help decision-makers prioritize healthcare interventions that improve nurse retention over healthcare interventions that reduce it or leave it unchanged, helping to reduce the nursing shortage. This review aims to inform the choice of intention-to-stay instrument to use in such studies, but the currently available information is insufficient to recommend one instrument over the others. A comparative study between instruments, including their predictive value for retention, would add substantially to the current knowledge base.

## Funding

Dutch Research Council, 406.XS.04.151. The funder was not involved in the review.

## Declaration of generative AI and AI-assisted technologies in the manuscript preparation process

During the preparation of this work the authors used ChatGPT and Grok in order to improve the writing. After using this tool/service, the authors reviewed and edited the content as needed and take full responsibility for the content of the published article.

## CRediT authorship contribution statement

**Myrthe van der Zanden:** Writing – review & editing, Writing – original draft, Visualization, Validation, Project administration, Methodology, Investigation, Formal analysis, Data curation, Conceptualization. **Saba Hinrichs-Krapels:** Writing – review & editing, Supervision, Methodology, Conceptualization. **Christa Niehot:** Writing – review & editing, Methodology. **Anna Teeuw:** Writing – review & editing, Conceptualization. **Swasti Madan:** Writing – review & editing, Data curation. **Naomi van der Linden:** Writing – review & editing, Writing – original draft, Visualization, Validation, Supervision, Project administration, Methodology, Investigation, Funding acquisition, Formal analysis, Data curation, Conceptualization.

## Declaration of competing interest

The authors declare the following financial interests/personal relationships which may be considered as potential competing interests:

Naomi van der Linden reports financial support was provided by Dutch Research Council. If there are other authors, they declare that they have no known competing financial interests or personal relationships that could have appeared to influence the work reported in this paper.

## Appendix Supplementary materials

Image, application 1
