# Supplementary material for: Instruments to measure nurses' intention-to-stay in the profession: A systematic literature review
Source: Int J Nurs Stud Adv. 2026 Jan 26;10:100496. doi: 10.1016/j.ijnsa.2026.100496 (PMC12905783; doi:10.1016/j.ijnsa.2026.100496)
Supplement: Supplementary file 1 [file mmc1.docx]

**Medline**

(exp "Nurses"/ OR exp "Nursing Staff"/ OR (nurse* OR ((nursing) ADJ6 (workforce* OR staff* OR personnel*))).ab,ti,kf.) **AND** (exp "Personnel Turnover"/ OR (((turnover* OR turn-over* OR stay* OR leave* OR quit*) ADJ3 (intent*)) OR ((personnel* OR employee* OR staff* OR nurse* OR anticipat*) ADJ3 (retent* OR turn-over* OR turnover*)) OR resign* OR attrition*).ab,ti,kf.) **AND** (exp "Surveys and Questionnaires"/ OR (question* OR survey* OR instrument OR instruments OR tool*).ab,ti,kf.) **NOT** ((news OR congres* OR abstract* OR book* OR chapter* OR dissertation abstract*).pt. AND 1800:2021.(sa_year))

**Embase**

('nurse'/exp OR 'nursing staff'/exp OR (nurse* OR ((nursing) NEAR/6 (workforce* OR staff* OR personnel*))):ab,ti,kw) **AND** ('turnover intention'/exp OR 'attrition'/exp OR 'turnover rate'/de/mj OR (((turnover* OR turn-over* OR stay* OR leave* OR quit*) NEAR/3 (intent*)) OR ((personnel* OR employee* OR staff* OR nurse* OR anticipat*) NEAR/3 (retent* OR turn-over* OR turnover*)) OR resign* OR attrition*):ab,ti,kw) **AND** ('questionnaire'/exp OR 'framework'/exp OR (question* OR survey* OR instrument OR instruments OR tool*):ab,ti,kw) **NOT** ([Conference Abstract]/lim AND [1800-2021]/py)

**Web of Science**

TS=((nurse* OR ((nursing) NEAR/5 (workforce* OR staff* OR personnel*))) **AND** (((turnover* OR turn-over* OR stay* OR leave* OR quit*) NEAR/2 (intent*)) OR ((personnel* OR employee* OR staff* OR nurse* OR anticipat*) NEAR/2 (retent* OR turn-over* OR turnover*)) OR resign* OR attrition*) **AND** (question* OR survey* OR instrument OR instruments OR tool*)) **NOT** (DT=(Meeting Abstract OR Meeting Summary) AND PY=1800-2021)

**Cochrane CENTRAL**

((nurse* OR ((nursing) NEAR/6 (workforce* OR staff* OR personnel*))):ab,ti,kw) AND ((((turnover* OR turn NEXT/1 over* OR stay* OR leave* OR quit*) NEAR/3 (intent*)) OR ((personnel* OR employee* OR staff* OR nurse* OR anticipat*) NEAR/3 (retent* OR turn NEXT/1 over* OR turnover*)) OR resign* OR attrition*):ab,ti,kw) AND ((question* OR survey* OR instrument OR instruments OR tool*):ab,ti,kw)

**PsycINFO**

(exp Nurses/ OR (nurse* OR ((nursing) ADJ6 (workforce* OR staff* OR personnel*))).ab,ti.) AND ((((turnover* OR turn-over* OR stay* OR leave* OR quit*) ADJ3 (intent*)) OR ((personnel* OR employee* OR staff* OR nurse* OR anticipat*) ADJ3 (retent* OR turn-over* OR turnover*)) OR resign* OR attrition*).ab,ti.) AND (exp surveys/ OR exp questionnaires/ or mail surveys/ OR (question* OR survey* OR instrument OR instruments OR tool*).ab,ti.) NOT ((news OR congres* OR abstract* OR book* OR chapter* OR dissertation abstract*).pt. AND 1800:2021.(sa_year))

**CINAHL**

(MH "Nurses+" OR MH "Nursing Staff, Hospital" OR MH "Registered Nurses by Specialty+" OR TI(nurse* OR ((nursing) N6 (workforce* OR staff* OR personnel*))) OR AB(nurse* OR ((nursing) N6 (workforce* OR staff* OR personnel*)))) **AND** (MH "Personnel Turnover" OR TI(((turnover* OR turn-over* OR stay* OR leave* OR quit*) N3 (intent*)) OR ((personnel* OR employee* OR staff* OR nurse* OR anticipat*) N3 (retent* OR turn-over* OR turnover*)) OR resign* OR attrition*) OR AB(((turnover* OR turn-over* OR stay* OR leave* OR quit*) N3 (intent*)) OR ((personnel* OR employee* OR staff* OR nurse* OR anticipat*) N3 (retent* OR turn-over* OR turnover*)) OR resign* OR attrition*)) **AND** (MH "Surveys+" OR MH "Questionnaires+" OR TI(question* OR survey* OR instrument OR instruments OR tool*) OR AB(question* OR survey* OR instrument OR instruments OR tool*))

**Google Scholar**

'intent to stay|leave|resign' nurse|nurses questionnaire|questionnaires|tool|tools|survey|surveys|tool|tools
